# Human Communication Dynamics in Digital Footsteps: A Study of the Agreement between Self-Reported Ties and Email Networks

**DOI:** 10.1371/journal.pone.0026972

**Published:** 2011-11-17

**Authors:** Stefan Wuchty, Brian Uzzi

**Affiliations:** 1 Northwestern Institute on Complex Systems and Network Science (NICO), Northwestern University, Evanston, Illinois, United States of America; 2 Kellogg School of Management and McCormick School of Engineering, Northwestern University, Evanston, Illinois, United States of America; Umeå University, Sweden

## Abstract

Digital communication data has created opportunities to advance the knowledge of human dynamics in many areas, including national security, behavioral health, and consumerism. While digital data uniquely captures the totality of a person's communication, past research consistently shows that a subset of contacts makes up a person's “social network” of unique resource providers. To address this gap, we analyzed the correspondence between self-reported social network data and email communication data with the objective of identifying the dynamics in e-communication that correlate with a person's perception of a significant network tie. First, we examined the predictive utility of three popular methods to derive social network data from email data based on volume and reciprocity of bilateral email exchanges. Second, we observed differences in the response dynamics along self-reported ties, allowing us to introduce and test a new method that incorporates time-resolved exchange data. Using a range of robustness checks for measurement and misreporting errors in self-report and email data, we find that the methods have similar predictive utility. Although e-communication has lowered communication costs with large numbers of persons, and potentially extended our number of, and reach to contacts, our case results suggest that underlying behavioral patterns indicative of friendship or professional contacts continue to operate in a classical fashion in email interactions.

## Introduction

Shifts in human communication have raised new questions about how role relationships -friendship and professional ties - are expressed in novel, electronic communication. Yet, as digital communication increasingly expands, research emphasizes that the object of ultimate interest is not the full set of contacts a person communicates with, but the identification of the social network of contacts where proprietary resources flow [1]. Current work attempting to define the social network within the flow of communication is based on the use of nodal demographic characteristics [2] to suppose the presence of likely ties or on flow thresholds for converting continuous email transmissions to binary yes/no friendships [3], [4], [5]. Despite the development of sophisticated tools [6] little empirical evidence exists on the strength of the correspondence between self-reported social ties and actual communication dynamics [1], [6], [7], [8], [9], [10], [11], suggesting that such knowledge could help advance research across disciplines [1], [2], [3], [4], [5], [6], [7], [8], [10], [11], [12], [13].

**Figure 1:**
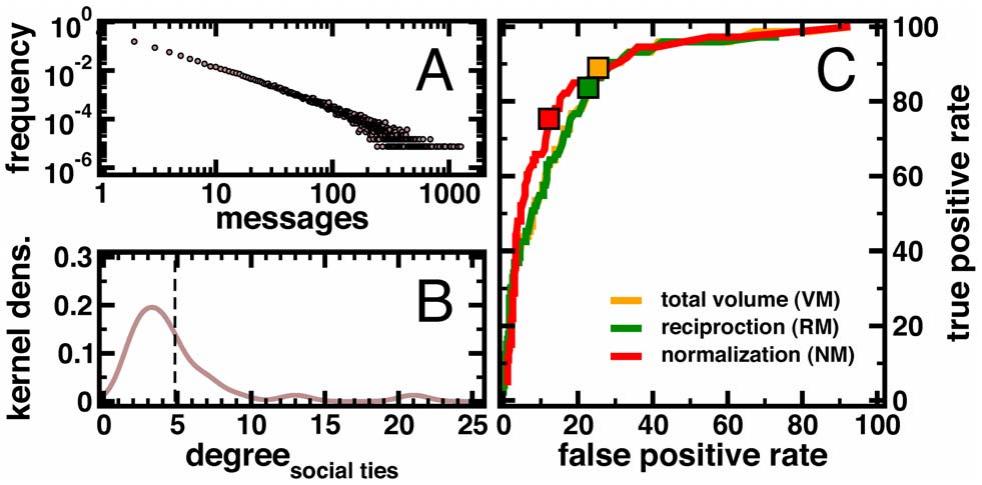
Data characteristics and ROC curves of conversion methods. (**A**) Counting the number of email messages that flow between a pair of persons we observed a strong power-law. (**B**) In the distribution of social ties a person is involved in, we found 4.8±3.8 contacts per person (dashed line). (**C**) Utilizing our methods, we converted emails to social attachments. ROCs indicate good agreement levels**,** comparing predicted ties to self-reported contacts (Area Under the ROC Curve (AUC) criterion: Normalization Method AUC = 0.891, Reciprocation Method AUC = 0.867, and Total Volume Method AUC = 0.867). Utilizing total volume method (VM), we indicated best TPR = 76.7% and FPR = 18.2% values that corresponded to the maximum correlation threshold between self-reported and predicted ties by a square. Analogously, we found TPR = 83.6% and FPR = 22.7 utilizing the reciprocation method (RM) and TPR = 75.3% and FPR = 12.2% using the normalization method (NM).

Here, we used self-reported human relations and email data from a typical professional services organization to investigate how email communication patterns map onto self-reported social network data. The significance of our approach lies in the ability to directly compare rare self-report and email data of the same sample population. Specifically, relative to previous work on managers' networks [11], [12], [14], [15], [16], [17], [18], our contributions include (1) the unique opportunity to compare self-reported social network data and email derived networks. (2) Highly detailed self-reported data. Our data allows for respondents to list up to 9 contacts whereas other work permits only 1–3 contacts (i.e., General Social Survey), and our respondents specified contacts as professional, social, or mentor ties for finer grained distinctions than normally permitted. (3) Also extending recent work, we do many robustness checks on the self-reported data to confirm as much as possible that the reported relationships are valid that previous work has not done. (4) We used to the fullest extent possible a second, large email dataset to confirm general patterns in the data. Nevertheless, while the data present one organization's network in detail, key descriptive statistics suggest that the relationships in this firm are not atypical relative to prior research. Specifically, we found that the volume of exchanged emails between contacts over our 6 month period of analysis follows a power law distribution (Fig. 1A), which is also typical of the distribution of email connections found in diverse settings [12], [14], [15], [16]. In the self-reported data, the average degree was roughly 5 (Fig. 1B), which is consistent with most recent work on self-reported networks [1], [3]. Finally, such distributions in self-reported and email data suggest that persons may communicate with many contacts, but only a handful of each person's contacts make up their “network.”

Methodologically, we used three different weighting methods to predict ties from email data. Building on these methods, we introduced a generic statistical adaption of these methods that can operate for estimating social networks from email data in populations where self-report data is unavailable. Finally, we tested if time-resolved information on email responsiveness, not heretofore examined in prior research, can have predictive utility in determining whether a tie is a social or professional connection in a social network.

## Results

### Agreement levels between Self-reported Social Network and Email Data

We tested three diverse methods for extracting key attachments from email that quantify the flow of emails between individuals in different ways. The volume method (VM) focuses on the volume of one-way email exchange and assumes that email volume over a threshold indicates a two-way social tie [4]. The reciprocation method (RM) scores a two-way tie by the geometric mean of exchanged emails [2]. The normalization method (NM) focuses on normalizing the strength of the email connections relative to the strongest link [19] while weaker links are scored according to a percentage of the strongest link (see Material and Methods for precise definitions of tie coding by method).

To assess these methods' general abilities to distinguish between self-reported and non-ties, we used the area under the Receiver Operating Characteristic curve (ROC) or (AUC) criterion [20]. We converted the email flow between pairs of individuals into binary yes/no relationships according to each method at specific thresholds. In Fig. 1C, we found that the normalization method provided the largest area under the ROC curve (AUC = 0.891) followed by the total volume and reciprocation method (AUC = 0.867).

While all methods showed a strong ability to find self-reported ties from email flows, the conversion of continuous scores into a binary web of relationships was based on arbitrary thresholds taken from past research. For example, Tyler et al. [4] set a threshold for converting continuous email data into a binary tie at a total volume of ≥30 messages, conditional on at least *5* reciprocated emails. In our analysis, such a threshold allowed a False Positive Rate FPR = 22.9% and a True Positive Rate TPR = 83.6%. Kossinets and Watts [2] considered a threshold for defining a binary tie if the geometric mean of their exchanged emails was at least 1, corresponding to a FPR = 68.8% and a TPR = 97.3% in our analysis. To find optimal thresholds, we rigorously quantified the agreement levels between predicted email ties and self-reported data. Specifically, we utilized Matthew's correlation coefficient between observed and predicted binary classifications. Since a coefficient of +1 represents a perfect prediction, 0 an average random prediction and −1 an inverse prediction we determined thresholds for each method that provided a maximum Matthew's coefficient. We found that the optimal threshold of RM provided the best True Positive Rate (TPR) (83.6%) and worst False Positive Rate (FPR) (22.7%); NM had the worst TPR (75.3%) and best FPR (12.2%), while VM had an intermediate TPR (76.7%) and FPR (18.2%).

On a topological level, we compared key network parameters, such as degree, clustering, betweenness, shortest paths and structural holes. We observed that these node-specific email network parameters correlated significantly with the corresponding measures observed in the self-reported data using standard Pearson's correlation coefficients (*p*<0.05, Table S1).

### Robustness checks

In a first robustness check we split our data in test and retest samples, randomly choosing ½ of the actual data and using Matthew's correlation coefficient to determine the optimal threshold for defining a binary network contact for each method. The Matthews correlation coefficient is the appropriate correlation for binary classifications because it takes into account true and false positives and negatives and is applicable in cases where the binary classes are of very different sizes. The Mathews correlation coefficient is in essence a correlation coefficient between observed and predicted binary classifications. It values are between −1 and +1. A coefficient of +1 represents a perfect prediction, 0 an average random prediction and −1 an inverse prediction. Subsequently, we applied each threshold to the remaining ½ of the data. Repeating these steps 1,000 times, we observed that the performance distributions compared well to unperturbed data (Fig. S1).

**Figure 2:**
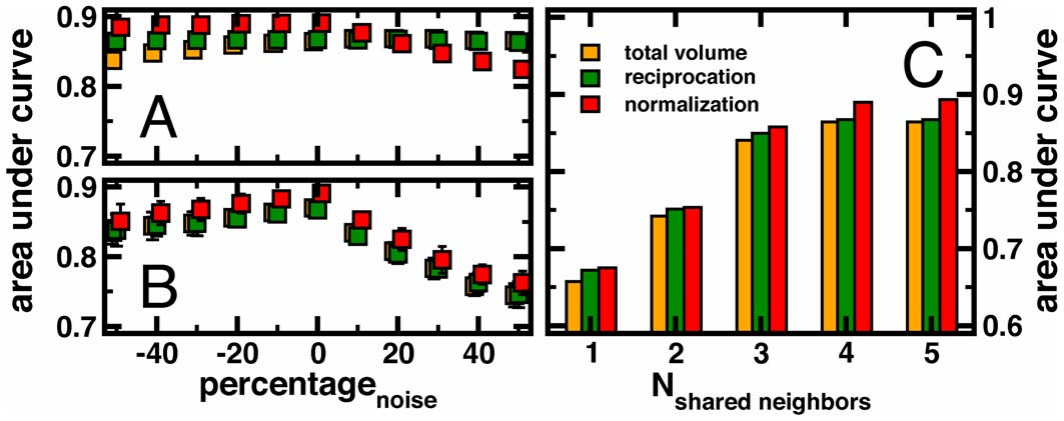
Noise robustness of conversion methods. In (**A**) we show results of validity tests of each method in the presence of measurement noise in the email data, where noise was simulated by randomly adding or deleting email messages in increments up to 50%. All the methods appear to be roughly robust to this type of measurement error. In (**B**) we randomly added false ties significantly lowering agreement. In (**C**) we randomly added self-reported ties, only if chosen persons share at least a certain number of common contacts as in real-life, recovering reliability.

In a different test we examined the sensitivity of our results toward measurement errors. In email, measurement error can occur if face-to-face communication is easier for some pairs of contacts than others. In self-reported surveys data measurement error can arise if respondents fail to report people or inadvertently indicate persons who are not in their network. Then the question of the internal validity of our analysis points to the error tolerance of the email and self-reported measures. Therefore, we gauged the error tolerance that can arise from the inadvertent inclusion or exclusion of links by progressively and randomly adding and deleting up to 50% of the emails from the actual data. Using ROC analysis, Fig. 2A indicates that all three methods are highly robust to this type of measurement error. Despite having up to 50% random error added or deleted from the email data, each method roughly maintains a steady area under the ROC curve. Showing the same experimental tests for the self-reported data in Fig. 2B, we found that the methods are robust to the random deletion of ties from the self-reported data as well. Conversely, randomly adding ties to the self-reported data significantly lowered the level of agreement in direct proportion to the percentage of false ties added to the network. However, we note that this test has weak experimental realism since persons rarely name fictive or random contacts in real life; rather individuals are increasingly likely to be tied to each other the more they share common third party contacts [2]. Therefore, a better test of robustness adds links only between persons who share third party contacts but who did not name each other as contacts in the self-report data. Therefore, we added ties between two randomly chosen individuals only if they shared at least a certain number of common contacts in the self-reported data. Fig. 2C indicates that the results are robust when false ties are added between persons who share increasing numbers of common 3^rd^ party contacts.

### Estimating a Social Network in Email Data without Self-reported Data

The above findings relied heavily on having both self-report and email data on the same sample. Since self-reported data is usually not available, we developed and tested a statistical null-model of email communication that could work hand-in-hand with the volume, reciprocation, and normalization methods. In this model, we compared the observed level of pairwise email flow to the expected pairwise level when email flow is randomized. Utilizing our conversion methods, we calculated intensity scores for each *i*–*j* link in the observed email under the guidelines of the volume, reciprocation, and normalization methods as previously described. For all pairs of individuals, *ij*, we counted all *n_ij_* emails that were sent from *i* to *j*. After randomly redistributing *n_ij_* among all pairs, we calculated the corresponding random intensity scores for each link. Repeating the randomization steps 10,000 times, we calculated means and standard deviations of random intensity scores and determined a *p*-value of each link using a *Z*-test.

Using ROCs as our agreement metric, we obtained an AUC = 0.838 using the normalization method (denoted *r*NM) with a statistically derived threshold, an AUC = 0.815 with the total volume method (*r*VM) and an AUC = 0.825 with the reciprocation method (*r*RM). These relatively good levels of agreement were further observed when we calculated network level topological parameters from the pairwise data for each method. Defining a tie between *i* and *j* if FDR<0.001 [21], we observed that the same topological parameters of social networks noted in the previous section compared well to the corresponding measures calculated directly from self-reported tie data (*p*<0.05, Table S2).

In a test-retest analysis, we calculated means and standard deviations of random exchange intensity scores in the first ½ of data and determined observed scores in the remaining ½ of the data. Using a *Z*-test, we obtained *p*-values of links in the retest samples and defined a tie if FDR<0.001. Results obtained with permutated statistical threshold method indicated good reliability as well (Fig. S2A), suggesting that our statistical model allows a reasonable approximation of (unknown) self-reported ties in this sample.

To test the sensitivity of these findings to measurement errors, we added and excluded up to 50% of the emails and self-reported ties as previously described. We found that the *r*NM, *r*VM, and *r*RM methods were fairly reliable to random measurement errors (Fig. S2BC), suggesting that the empirical thresholds are related to standard statistical thresholds for distinguishing observed behavioral patterns from simple random interaction. At least in this data sample, we conclude that these statistical patterns can be used as a proxy for a valid threshold when no self-reported data are available.

### Response Time Dynamics and Classification of Different Types of Ties

Considering dynamic aspects, we drew on sociological theory to understand the factors besides volume and reciprocation of exchanges that can discriminate relationships from non-relationships. Social theory holds that the closer the social relation, the more responsive persons are to each other's desire for attention, prompting them to reply more quickly [16], [18]. Considering such communication dynamics, we examined whether response times differed for self-reported and non-self-reported relationships.

**Figure 3:**
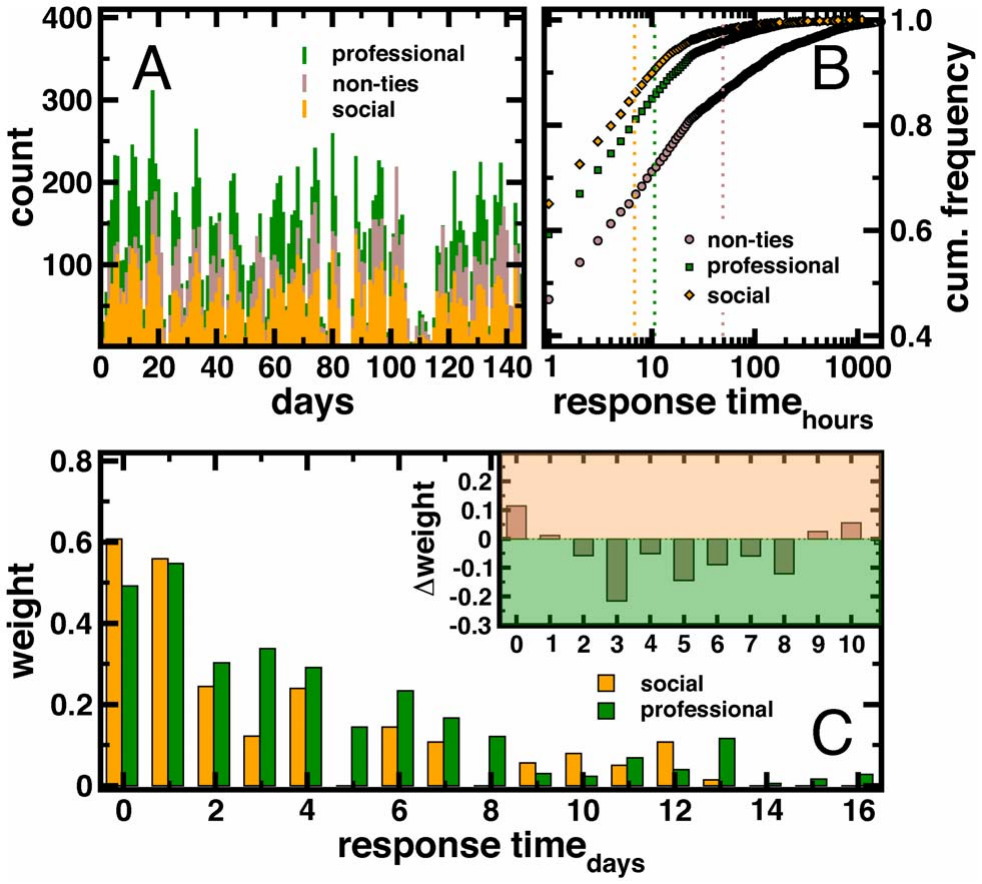
Time-resolved data characteristics. In (**A**) we show a profile of emails that were exchanged along social and professional ties. Non-ties referred to pairs of individuals that exchanged emails but were not self-reported as contacts. (**B**) Frequency distributions of response times (mean  =  dotted lines) of different types of ties indicate that social friendship ties had the lowest response time. (**C**) Discriminating between self-reported ties and non-ties using the normalization method, we determined the weight of emails that were exchanged in a certain time interval. We observed that emails sent within the same day contributed significantly to the discrimination of social and professional ties from non-ties. Determining weight difference, emails exchanged in short intervals predominantly characterize social friendship ties while emails that were sent with up to 8 days delay had a higher impact on the discrimination of professional ties (inset).

Examining differences across types of ties we found that the highest volumes of communication occurred along the weakest of social relationships (Fig. 3A): professional (including professional and mentoring ties) and non-ties both were associated with a higher absolute aggregate volume of email communication than social friendship ties. Examining if response time indicates differences in the pattern of communication for different types of ties, we grouped emails into bins according to the time interval between consecutively exchanged emails between *i* and *j*. Considering daily resolution of the elapsed time until a response occurred, *i*'s email ends up in bin Δ*t* = 0 if *i* received an email from *j* and responded within 24 hours. Fig. 3B indicates clear differences in the cumulative frequencies of emails in time resolved bins that have been sent along social, professional, and non-ties. Despite lower absolute volume along social ties, we observed that social closeness is indeed positively associated with response time. Socially tied individuals appear to communicate less frequently with each other but respond to each other more quickly when an email is received. Checking the statistical significance of such results we resorted to a random background model. In particular, we randomly distributed emails in time and determined the average response times along social, professional and non-ties. Averaging over 100 randomizations, we obtained a mean average response time of 6.5±0.1 hours while we observed an average response time of 6.8 hours (dotted line in Fig. 3B) along social ties in the unperturbed email data. Analogously, we obtained a mean of 12.2±0.1 hours in the random model and 10.8 hours along professional ties. Along non-ties, we observed an expected average response time of 49.2 hours and obtained 71.1±0.3 hours in the random model, suggesting that the distribution of interval times along self-reported ties are non-random.

Because communication patterns in organizations can be affected by local incentives and norms for responsiveness, we performed the same analysis on an independent population that included all the email transmissions among 600 MBA students for a two-year period. Furthermore, the MBA data allows a crude classification of students' bilateral relationships as social or professional contacts. Students who were enrolled in the same extracurricular activity (e.g., soccer team, wine tasting club, cello club, etc.) but were not enrolled in the same classes were coded as friends, while students enrolled in the same classes but not in extracurricular activities were coded as professional contacts. Using this crude classification, we found remarkably similar patterns between response times and types of contacts in such a social network (Fig. S3).

To formally test whether response time was a statistically significant classifier of types of ties, we performed a discriminant analysis. First, we calculated email exchange scores in daily bins, representing each pair of individuals by a vector of bin-specific scores (see Materials and Methods). Second, we used the I-Relief algorithm [22], [23], which provides a “weight,” a quantitative value, indicating the importance of emails in bins of response time Δ*t* for discriminating social from professional ties. Weights are on a scale of 0.0 to some positive number, where 0.0 indicates that the time interval provides no discriminating impact. In Figs. 3C and S4 we found that rapid response times contribute to the accurate distinction of both self-reported social and professional ties from non-ties. Shortest time intervals especially characterized social ties (Fig. 3C) while longer response times were strongly indicative of professional ties.

Having found that dynamic response time information has predictive utility for classifying ties as social or professional, we returned to our original three methods of deriving contacts from email data. In particular, we wondered whether the introduction of response time information would increase their ability to accurately detect a person's social network within their email communication. To classify ties based on an original conversion method *plus* the response time information, we used hourly time intervals Δ*t* as variables of each (non-)tie, representing each link by a vector of hourly time-resolved scores. To account for the aggregate effects of response time information in the prediction of self-reported relationships, we used the random forest algorithm [24]. This ensemble-learning method repeatedly draws a bootstrap sample from the underlying data and constructs decision trees with a random subset of variables to separate statistical relationships from spurious ones. In our case, we constructed 10,000 trees where we randomly sampled ⅔ of the data and 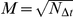 out of all *N* time intervals Δ*t* and used the remaining ⅓ of the data to test the performance of each decision tree. Considering that each pair occurs several times in such test sets, the method reports the fraction of times the pair of partners was classified correctly as a self-reported tie. To observe the best agreement between predicted and self-reported ties, we determined the fraction with a maximum Matthew's correlation coefficient at a FPR<15%, curbing identification errors since false positives relate to a larger absolute number of ties (Fig. S5A). We found that all measures of an ego network structure calculated from the self-reported data correlated significantly (*p*<0.05, Table S3) with the email derived, time resolved networks. Performing a test-retest analysis, we randomly split emails, trained our time-resolved methods on the test sets and checked the performance on the retest sets, allowing us to find fairly stable results (Fig. S5A). Furthermore, time resolved data also offered stable results if large amounts of noise were introduced in the underlying email and self-reported data as previously described (Fig. S5BC).

## Discussion

We used a rare combination of all exchanged email and self-reported social network data on a subset of all the individuals at a large company in one establishment to investigate our ability to map social networks from email transmissions. We found that the bilateral volume and reciprocal flow of emails measured by three different methods are proxies for social ties, suggesting that contemporary e-communication has not yet drastically changed fundamental patterns of human interaction [7], [16], [25]. Despite the fact that e-communication has lowered the cost of communication as well as barriers to communicate over long distances, historic communication and behavioral patterns that have defined friendship or professional contacts continue to operate in comparable ways in face-to-face and online interactions.

Another contribution of our work shows that response time plays a key role in separating socially close and distant contacts while differences in the predictive utility of our three methods with and without the incorporation of time resolved data were minor. If the classification objective is to derive the “social network” from the communication network, where the social network is a consolidation of different types of important personal relations, time resolved response data does not appear to be critical for the categorization of ties. Only when the objective is the distinction between types of ties within the social network, short response times largely characterize close social relationships, an observation that may have implications for the quality of feedback, resource mobilization, group decisions, and other important patterns of collective human behavior.

Several broad limitations of research on social and e-communication networks may be noteworthy in our work and for future studies. (i) Although we observed a promising link between email flows and social ties, our methods cannot capture unobservable characteristics within an organization that may not generalize to other settings. (ii) Due to the general sparsity of social networks a high true positive and a low false positive rate can nonetheless produce numerous identification errors because false positive rates relate to a larger absolute number of ties. (iii) Since a social tie is a binary variable quantification must rely on the accuracy of persons' perception in paper and pencil surveys or on the assignment of valid, numerical thresholds [17], making error in dichotomization inevitable.

To mitigate these limitations, we took extensive precautions to insure that our survey instrument was designed validly and reliably on all key design issues related to accurate recall and truncation [17], [26], [27] (See Methods and Materials). If truncation had taken place, most respondents would have reported a number of contacts equal to or close to the maximum permitted by the survey. However, the average was 50% of the total of contacts that could be named, a reasonable subset that is in line with past research on intra-organizational networks [12], [14], [15], [16]. For example, Eagle et al. [3] found that the 91 college students in their study only named 1.3 others out of the possible 91 as “friends.” Christakis and Fowler's [1] longitudinal study had a mean number of contacts of <2. Also, we gauged the error tolerance of reporting errors by the random inclusion and exclusion of ties and emails, allowing us to find that our methods are largely robust to this sort of measurement error. Consistent with past research on network sampling [28] we conclude that random noise primarily creates new links that have only a nominal level of intensity or severs existing links that have nominal intensity. Thus, links that are characterized by a relatively intensive level of email exchange, *i.e.*, potential social attachments, are likely to be robust to noise in email activity. With regard to testing our work on other data, we were able to confirm some of the findings in a second dataset.

As e-communication channels and social media use increases, we view this study as providing a necessary first step to the understanding of the correspondence between the social and the communication network, indicating how different types of relationships and digital communication behavior are related. Of key importance is the understanding that e-communication mirrors patterns of face-to-face communication in regard to different types of relationships while the availability of electronic communication channels drastically reduced communication costs and extended our potential number of and reach to contacts, the email dynamics we observed suggests that fundamental patterns of friendship and professional connections continue to operation in their classical fashion. How these dynamics aggregate to change human dynamics is putatively dependent on the contextual basis of our findings. Therefore, a critical question for future work is to examine how these patterns may operate in other contexts and in an increasingly expanding universe of digital communication.

## Materials and Methods

### Email and Self-reported Data

Our primary email data included all 1,493,441 internal, non-distribution list emails sent between July 2006 and January 2007 by all 1,052 managers of a typical professional services company that offers various forms of consulting services to clients. The name, location, and specific sector of the company are omitted, and email data did not contain any text or subject information for purposes of confidentiality in the analysis. Furthermore, group emails that were sent to more than one person and external emails were excluded for privacy reasons.

Like other professional services firms, the managers of this company deal with clients from other companies who approach them with their organizational problems. When engaging with clients, managers work together with other managers and staff in the organization who provide them with professional resources needed to service clients and develop new business. The social network survey data came from all 31 managers in a regional office of the company. The questionnaire was developed in accordance with standard, pilot tested methods [1], [12], [15], [25], [26], [27], [28] to determine the accurate size of managers' networks and had 100% response rate. Respondents named up to 9 ties, which was meant to identify contacts a respondent had at the firm. Respondent could further code contacts as professional, social friendship, and mentor attachments. Professional ties provided work related information, social ties were colleagues seen outside of work and mentor ties were persons who provided private professional advice. As a contact respondents could name anyone in the whole world-wide organization. Despite the observation that two respondents named persons outside their branch office the inclusion or exclusion of such respondents did not change the reported results. In particular, the questionnaire provided 20.2% social ties and 79.8% professional ties. For all network- generating items in the survey, aided recall was used, a technique that presents a pick list of all possible company contacts to each respondent. Aided recall is a widely considered standard method in paper and pencil social network questionnaires that has been shown to increase accuracy of responses. In particular, the company designed the questionnaire, collected, archived and anonymized the data with randomized ID#s before we obtained the data. We neither had interactions with any of the subjects nor intervened with the survey for the purpose of our research. IRB exempts data collections when (i) all data are anonymized, (ii) there is no interaction with subjects, and (ii) all data are archival. We received verbal confirmation that written informed consent was received by the company from each participant using the email system. We did not seek written content because the study is IRB exempt, and written consent was unduly expensive for the company to retrieve from their archives.

Our second email dataset captures content free e-mail logs for the duration of 1 year (∼11.5 million e-mails; ∼4.5 million student-student e-mails) among two cohorts of full-time MBA students at a top MBA program (mean GMAT >90^th^ percentile, cohort size ∼550 students). Students are randomly assigned to sections within the school, minimizing selection effects. Observations began when students first met each other, eliminating censoring. E-mail log data was stripped of content and subject headings. Any identifying information were combined with information from the university office of registration to determine students who shared the same class or were involved in the same shared activities, identifying 86% as professional and 14% as social ties. An independent 3^rd^ party at the university anonymized and combined all data before we received it. No personally identifiable information was handled by the researchers. Because the university did not provide any personally identifiable information, student consent was not sort out per FERPA requirements. The above protocols were conducted under IRB project # STU00002048.

### Email conversion methods

In the total volume method (VM), we calculated the total number of emails *N* that were exchanged between nodes *i* and *j* as 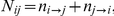 where 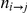 is the number of emails sent from *i* to *j*, and demanded that both 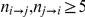 [4]. In the reciprocation method (RM), we defined the strength of a tie as 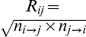 [2]. In the normalization method (NM), we modeled the strength of a tie as
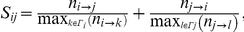
where Γ*_i_* is the set of contacts of person *i*. Assuming that *i* sent *80%* of its email to *j* and *20%* of its email to *k,* the *i*–*j* link has a value of 1 (*i.e.* 0.80/0.80) while the *i*–*k* link has a value of 0.25 (*i.e.* 0.20/0.80) from *i*'s perspective. Since the value of a tie from both actor's perspectives is the sum of their respective one-way values, the link between *i* and *j* had a value of 2 (*i.e.* 1.0+1.0) if *i* and *j* were both each other's strongest ties. If *j* was *i*'s strongest link but *j* did not send any emails to *i*, the *i*–*j* link had a value of 1 (*i.e.* 1.0+0.0) and *vice versa*. Therefore, the NM method places the strength of each tie in an interval between a lower (*i.e.* 0) and an upper bound (*i.e.* 2) while the VM and RM methods have lower but no upper bound.

### ROC curves

In comparing email ties to self-reported ties we defined true positive (*TP)*, true negative (*TN*), false positive (*FP*) and negative hits (*FN*). Determining the performance of our models we considered several measures: to construct ROC curves, we defined true positive rate as 
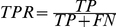
 and false positive rate as 
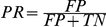
 . Matthew's correlation coefficient was defined as 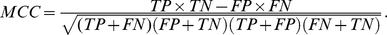


### Measures of Network Characteristics

We defined structural holes as a node specific measure by 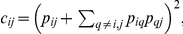 \ where *p_ij_* is the proportion of *i*'*s* relations invested in contact *j* [15], [29]. The total in parentheses is the proportion of *i*'*s* relations that are directly or indirectly invested in connection with contact *j*. A low value of structural holes essentially points to the nodes role as a connector of different network parts, therefore serving as a gateway of information flow between different, densely connected areas of the total network. In turn, a high value indicates that a node is predominantly surrounded by peers in a densely connected part of the network.

We defined the clustering coefficient as the fraction of actual links among all neighbors of a node *I*, defined as 
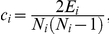
 where *E_i_* is the number of links between *N_i_* contacts of node *i* [30].

Betweenness centrality reflects a nodes appearance in shortest paths through the whole network. Specifically, we defined betweenness centrality as 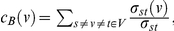 where σ*_st_* is the number of shortest paths between nodes *s* and *t* while *σ_st_ (v)* is the number of shortest paths running through *v* [31].

### Time Resolved Conversion Methods

Utilizing hourly or daily resolution of the time that elapsed until an individual responds to an email from the other person we calculated a time dependent score. Specifically, we adapted the definition of the normalization score and calculated a time resolved score between individuals *i* and *j* as 
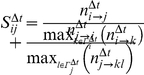
 where 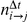 is the number of emails *i* sent to *j*, and 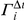 is the total number of emails that *i* sent within a time interval Δ*t.* Each (non-)tie between *i* and *j* was represented by a vector, holding all time dependent scores 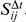 Similarly, we defined time resolved scores with the reciprocation method as 
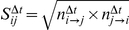
 and modeled the time resolved score of the total volume method as 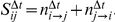


### I-Relief algorithm

To identify the contribution of single time intervals Δt to distinguish types of ties, the I-Relief algorithm [23] defines sets of nearest hits *H_n_* (self-reported tie) and nearest misses *M_n_* (non-tie). The objective function of the algorithm is to scale each feature (*i.e.* time interval) such that the average margin in a weighted feature space is maximized. Briefly, the I-Relief algorithm estimates probability distributions of the unobserved data as exponential functions *f(d) = e^-d/s^* where we set *s* = 2. Iteratively, I-Relief adopts a quasi Expectation-Maximization strategy to assess the weights of the underlying features until convergence is reached.

### Random Forests

Random Forests [24] is an ensemble learning method where each decision tree is constructed using a different bootstrap sample of the data (‘bagging’). In addition, random forests change how decision trees are constructed by splitting each node, using the best among a subset of predictors randomly chosen at that node (‘boosting’). Compared to many other classifiers this strategy turns out to be robust against over-fitting, capturing aggregate effects between predictor variables.

In more detail, classification performed by random forests is based on three steps: (i) *N* bootstrap samples are drawn from the underlying data. For each of the bootstrap samples, an un-pruned decision tree is constructed where at each node *M* predictors are randomly sampled and (ii) the best split from those variables is finally picked. (iii) New data is predicted by aggregating the predictions of *N* trees. For each decision tree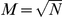 out of all *N* variables and ⅔ of the data was sampled. The remaining ⅓ of the data (*i.e* out-of bag examples) was used as a cross-validation set to test the classification performance of the underlying decision tree.

## Supporting Information

Figure S1 **(A)** Utilizing the total volume method, we converted emails to social attachments. In the ROC, we indicated best FPR = 18.2% and TPR = 76.7% (dashed orange lines). Testing the robustness of our methods by randomly splitting the email transmissions into a “test” and “retest” set, we found a mean FPR = 18.7±4.4 and a mean TPR = 74.5±8.0. In the inset, we found best FPR = 22.7% and TPR = 83.6% utilizing the reciprocation method (dashed green lines). After a test-retest analysis, the reciprocation method provided a mean FPR = 20.7±5.6 and a mean TPR = 77.1±9.2. **(B)** Using the normalization method, we found a mean FPR = 10.7±3.0 and a mean TPR = 74.5±8.0 in a test-retest step, results that correlate well with the best FPR = 12.2% and TPR = 75.3%.
(PDF)

Figure S2 **(A)** shows ROC curves for three email-to-social network conversion methods using permuted email data. Utilizing the total volume method we found a FPR = 17.2% and TPR = 74.7% while the reciprocation method yielded a FPR = 22.1% and FPR = 80.0%. Finally, the normalization method allowed us to find a FPR = 12.1% and a TPR = 76.0%. Performing a test-retest analysis, we randomly split emails, trained our permuted methods on the test sets and checked the performance on the retest sets. Utilizing the normalization method we found a mean FPR = 17.6±0.7 and mean TPR = 73.5±2.1 while the reciprocation method yielded a mean FPR = 19.5±0.7 and a mean TPR = 76.7±2.0. Finally, the normalization method allowed us to find a mean FPR = 12.7±0.6 and a mean TPR = 74.0±2.1. In **(B)** we show results of tests of the validity of each method in the presence of measurement noise in the email data, where noise was simulated by randomly adding or deleting email messages at random times in increments up to 50%. In **(C)**, we repeated the procedure for adding self-reported ties, if chosen persons shared at least a certain number of common contacts.
(PDF)

Figure S3 We display the frequency distribution of response times for different types of ties utilizing email transmissions among more than 500 MBA students over a 2-year period of time. Specifically, we only accounted for time intervals of <1,000 hours. We conclude that social ties have shorter response times than professional ties.
(PDF)

Figure S4 Analogously to Fig. 3C in the main paper we utilized the total volume and reciprocation method (inset) and found that emails with a short response-time significantly contributed to the difference between social self-reported and other ties.
(PDF)

Figure S5 **(A)** shows ROC curves for three email-to-social network conversion methods that utilize time-resolved data. Utilizing the total volume method we found a FPR = 13.6% and TPR = 66.7% while the reciprocation method yielded a FPR = 14.9% and FPR = 58.7%. Finally, the normalization method allowed us to find a FPR = 10.5% and a TPR = 69.3%. Performing a test-retest analysis, we randomly split emails, trained our permuted methods on the test sets and checked the performance on the retest sets. Utilizing the total volume method we found a mean FPR = 11.2±2.9 and a mean TPR = 56.1±10.4 while the reciprocation method yielded a mean FPR = 12.2±3.0 and a mean TPR = 57.4±8.8. Finally, the normalization method allowed us to find a mean FPR = 10.8±2.8 and a mean TPR = 60.8±6.6. In **(B)** we show results of tests of the validity of each method in the presence of measurement noise in the email data, where noise was simulated by randomly adding or deleting email messages at random times in increments up to 50%. In **(C)**, we repeated the procedure for adding self-reported ties, if chosen persons shared at least a certain number of common contacts.
(PDF)

Table S1 We show Pearson's correlations between a person's self-reported and email derived network characteristics for the 31 partners in the same office, utilizing total volume (VM), reciprocation (RM) and normalization method (NM).
(PDF)

Table S2 We show Pearson's correlations between a person's self-reported and email derived network characteristics for the 31 partners in the same office, utilizing the randomized total volume (rVM), reciprocation (rRM) and normalization method (rNM).
(PDF)

Table S3 We show Pearson's correlations between a person's self-reported and email derived network characteristics for the 31 partners in the same office, utilizing the time-resolved total volume (tVM), reciprocation (tRM) and normalization method (tNM).
(PDF)
