# Supplementary material for: Human Communication Dynamics in Digital Footsteps: A Study of the Agreement between Self-Reported Ties and Email Networks
Source: PLoS One. 2011 Nov 17;6(11):e26972. doi: 10.1371/journal.pone.0026972 (PMC3219656; doi:10.1371/journal.pone.0026972)
Supplement: Figure S1 — (A) Utilizing the total volume method, we converted emails to social attachments. In the ROC, we indicated best FPR = 18.2% and TPR = 76.7% (dashed orange lines). Testing the robustness of our methods by randomly splitting the email transmissions into a “test” and “retest” set, we found a mean FPR = 18.7±4.4 and a mean TPR = 74.5±8.0. In the inset, we found best FPR = 22.7% and TPR = 83.6% utilizing the reciprocation method (dashed green lines). After a test-retest analysis, the reciprocation method provided a mean FPR = 20.7±5.6 and a mean TPR = 77.1±9.2. (B) Using the normalization method, we found a mean FPR = 10.7±3.0 and a mean TPR = 74.5±8.0 in a test-retest step, results that correlate well with the best FPR = 12.2% and TPR = 75.3%. (PDF) [file pone.0026972.s001.pdf]

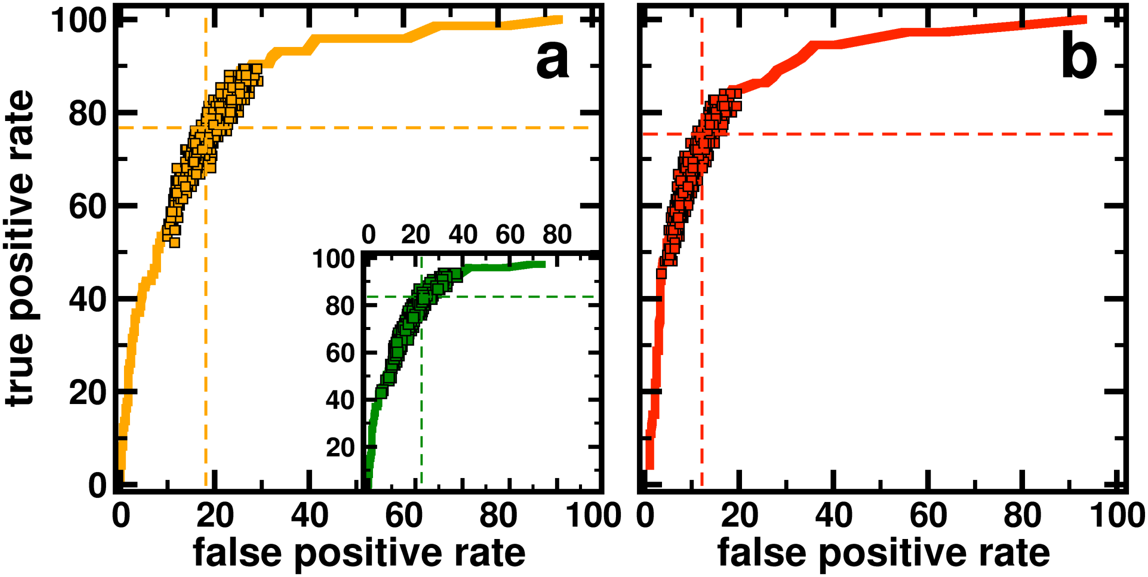

Figure S1: (A) Utilizing the total volume method, we converted emails to social attachments. In the ROC, we indicated best FPR = 18.2% and TPR = 76.7% (dashed orange lines). Testing the robustness of our methods by randomly splitting the email transmissions into a “test” and “retest” set, we found a mean FPR = 18.7 ± 4.4 and a mean TPR = 74.5 ± 8.0. In the inset, we found best FPR = 22.7% and TPR = 83.6% utilizing the reciprocation method (dashed green lines). After a test-retest analysis, the reciprocation method provided a mean FPR = 20.7 ± 5.6 and a mean TPR = 77.1 ± 9.2. **(B)** Using the normalization method, we found a mean FPR = 10.7 ± 3.0 and a mean TPR = 74.5 ± 8.0 in a test-retest step, results that correlate well with the best FPR = 12.2% and TPR = 75.3%.
