# Supplementary material for: Human Communication Dynamics in Digital Footsteps: A Study of the Agreement between Self-Reported Ties and Email Networks
Source: PLoS One. 2011 Nov 17;6(11):e26972. doi: 10.1371/journal.pone.0026972 (PMC3219656; doi:10.1371/journal.pone.0026972)
Supplement: Figure S2 — (A) shows ROC curves for three email-to-social network conversion methods using permuted email data. Utilizing the total volume method we found a FPR = 17.2% and TPR = 74.7% while the reciprocation method yielded a FPR = 22.1% and FPR = 80.0%. Finally, the normalization method allowed us to find a FPR = 12.1% and a TPR = 76.0%. Performing a test-retest analysis, we randomly split emails, trained our permuted methods on the test sets and checked the performance on the retest sets. Utilizing the normalization method we found a mean FPR = 17.6±0.7 and mean TPR = 73.5±2.1 while the reciprocation method yielded a mean FPR = 19.5±0.7 and a mean TPR = 76.7±2.0. Finally, the normalization method allowed us to find a mean FPR = 12.7±0.6 and a mean TPR = 74.0±2.1. In (B) we show results of tests of the validity of each method in the presence of measurement noise in the email data, where noise was simulated by randomly adding or deleting email messages at random times in increments up to 50%. In (C), we repeated the procedure for adding self-reported ties, if chosen persons shared at least a certain number of common contacts. (PDF) [file pone.0026972.s002.pdf]

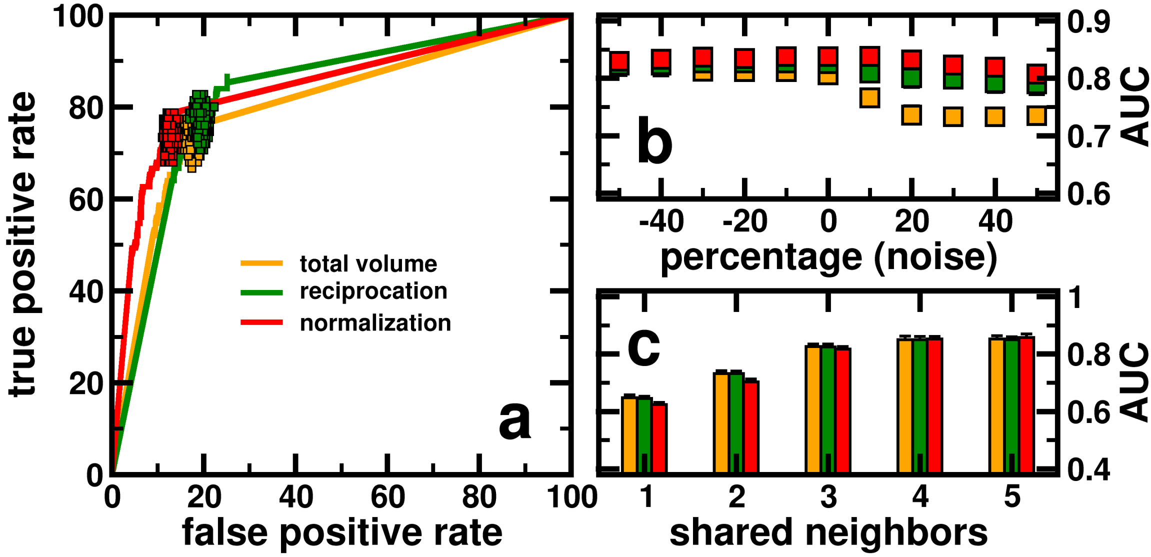

Figure S2: (A) shows ROC curves for three email-to-social network conversion methods using permuted email data. Utilizing the total volume method we found a FPR = 17.2% and TPR = 74.7% while the reciprocation method yielded a FPR = 22.1% and FPR = 80.0%. Finally, the normalization method allowed us to find a FPR = 12.1% and a TPR = 76.0%. Performing a test-retest analysis, we randomly split emails, trained our permuted methods on the test sets and checked the performance on the retest sets. Utilizing the normalization method we found a mean FPR = 17.6 ± 0.7 and mean TPR = 73.5 ± 2.1 while the reciprocation method yielded a mean FPR = 19.5 ± 0.7 and a mean TPR = 76.7 ± 2.0. Finally, the normalization method allowed us to find a mean FPR = 12.7 ± 0.6 and a mean TPR = 74.0 ± 2.1. In **(B)** we show results of tests of the validity of each method in the presence of measurement noise in the email data, where noise was simulated by randomly adding or deleting email messages at random times in increments up to 50%. In **(C)**, we repeated the procedure for adding self-reported ties, if chosen persons shared at least a certain number of common contacts.
