# Supplementary material for: Human Communication Dynamics in Digital Footsteps: A Study of the Agreement between Self-Reported Ties and Email Networks
Source: PLoS One. 2011 Nov 17;6(11):e26972. doi: 10.1371/journal.pone.0026972 (PMC3219656; doi:10.1371/journal.pone.0026972)
Supplement: Figure S3 — We display the frequency distribution of response times for different types of ties utilizing email transmissions among more than 500 MBA students over a 2-year period of time. Specifically, we only accounted for time intervals of <1,000 hours. We conclude that social ties have shorter response times than professional ties. (PDF) [file pone.0026972.s003.pdf]

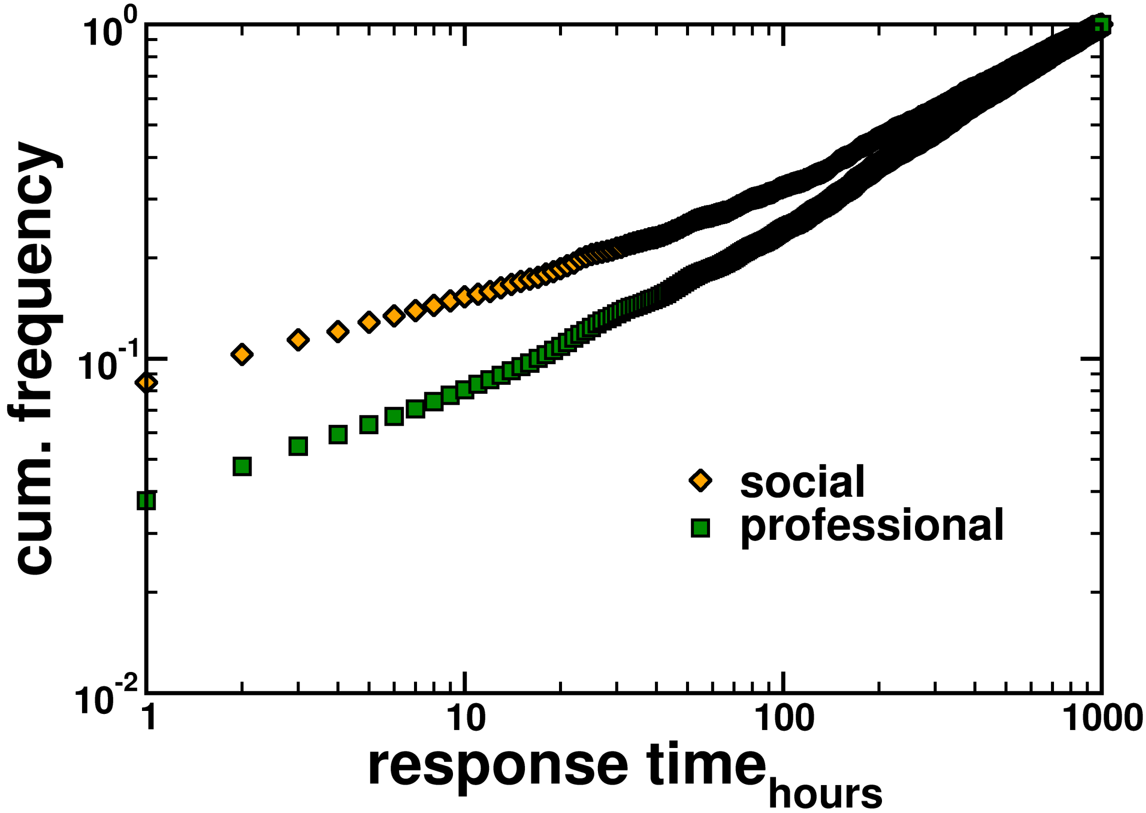

Figure S3: We display the frequency distribution of response times for different types of ties utilizing email transmissions among more than 500 MBA students over a 2-year period of time. Specifically, we only accounted for time intervals of < 1,000 hours. We conclude that social ties have shorter response times than professional ties.
