# Supplementary material for: Human Communication Dynamics in Digital Footsteps: A Study of the Agreement between Self-Reported Ties and Email Networks
Source: PLoS One. 2011 Nov 17;6(11):e26972. doi: 10.1371/journal.pone.0026972 (PMC3219656; doi:10.1371/journal.pone.0026972)
Supplement: Figure S4 — Analogously to Fig. 3C in the main paper we utilized the total volume and reciprocation method (inset) and found that emails with a short response-time significantly contributed to the difference between social self-reported and other ties. (PDF) [file pone.0026972.s004.pdf]

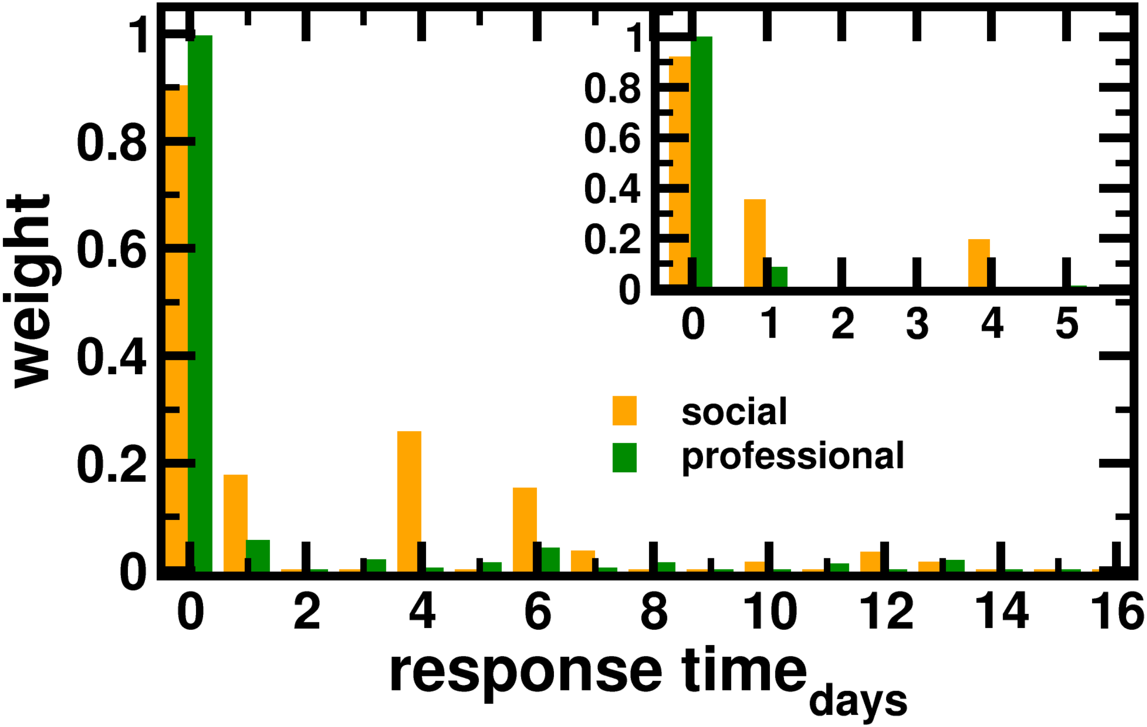

Figure S4: Analogously to Fig. 3C in the main paper we utilized the total volume and reciprocation method (inset) and found that emails with a short response-time significantly contributed to the difference between social self-reported and other ties.
