# Supplementary material for: Human Communication Dynamics in Digital Footsteps: A Study of the Agreement between Self-Reported Ties and Email Networks
Source: PLoS One. 2011 Nov 17;6(11):e26972. doi: 10.1371/journal.pone.0026972 (PMC3219656; doi:10.1371/journal.pone.0026972)
Supplement: Table S1 — We show Pearson's correlations between a person's self-reported and email derived network characteristics for the 31 partners in the same office, utilizing total volume (VM), reciprocation (RM) and normalization method (NM). (PDF) [file pone.0026972.s006.pdf]

Table S1: We show Pearson's correlations between a person's self-reported and email derived network characteristics for the 31 partners in the same office, utilizing total volume (VM), reciprocation (RM) and normalization method (NM).

N = 31	**total volume**	**reciprocation**	**normalization**
	**VM**	**RM**	**NM**
**degree**	**0.76*** **	**0.78*** **	**0.64*** **
**clustering**	**0.33***	**0.40***	**0.35***
**shortest path**	**0.36***	**0.40***	**0.38***
**betweenness**	**0.70*** **	**0.68*** **	**0.36***
**structural holes**	**0.47** **	**0.54*** **	**0.35***

*** P < 0.001, ** P < 0.01, * P < 0.05
