# Supplementary material for: Human Communication Dynamics in Digital Footsteps: A Study of the Agreement between Self-Reported Ties and Email Networks
Source: PLoS One. 2011 Nov 17;6(11):e26972. doi: 10.1371/journal.pone.0026972 (PMC3219656; doi:10.1371/journal.pone.0026972)
Supplement: Table S2 — We show Pearson's correlations between a person's self-reported and email derived network characteristics for the 31 partners in the same office, utilizing the randomized total volume (rVM), reciprocation (rRM) and normalization method (rNM). (PDF) [file pone.0026972.s007.pdf]

Table S2: We show Pearson's correlations between a person's self-reported and email derived network characteristics for the 31 partners in the same office, utilizing the randomized total volume (rVM), reciprocation (rRM) and normalization method (rNM).

N = 31	**total volume**	**reciprocation**	**normalization**
	**rVM**	**rRM**	**rNM**
**degree**	**0.72*** ***	**0.75*** ***	**0.64*** ***
**clustering**	**0.32***	**0.49***	**0.31***
**shortest path**	**0.38***	**0.36***	**0.37***
**betweenness**	**0.60*** ***	**0.60*** ***	**0.36***
**structural holes**	**0.48*** *	**0.51*** *	**0.34***

*** P < 0.001, ** P < 0.01, * P < 0.05
