# Supplementary material for: Human Communication Dynamics in Digital Footsteps: A Study of the Agreement between Self-Reported Ties and Email Networks
Source: PLoS One. 2011 Nov 17;6(11):e26972. doi: 10.1371/journal.pone.0026972 (PMC3219656; doi:10.1371/journal.pone.0026972)
Supplement: Table S3 — We show Pearson's correlations between a person's self-reported and email derived network characteristics for the 31 partners in the same office, utilizing the time-resolved total volume (tVM), reciprocation (tRM) and normalization method (tNM). (PDF) [file pone.0026972.s008.pdf]

Table S3: We show Pearson's correlations between a person's self-reported and email derived network characteristics for the 31 partners in the same office, utilizing the time-resolved total volume (tVM), reciprocation (tRM) and normalization method (tNM).

N = 31	**total volume**	**reciprocation**	**normalization**
	**tVM**	**tRM**	**tNM**
**degree**	**0.81*** **	**0.68*** **	**0.71*** **
**clustering**	**0.36***	**0.38***	**0.32***
**shortest path**	**0.54*** **	**0.38***	**0.46** **
**betweenness**	**0.80*** **	**0.48** **	**0.50** **
**structural holes**	**0.37***	**0.36***	**0.40** **

*** P < 0.001, ** P < 0.01, * P < 0.05
